# Symptoms and experiences of frailty in lung cancer patients with chemotherapy: A mixed-method approach

**DOI:** 10.3389/fonc.2022.1019006

**Published:** 2022-10-06

**Authors:** Liran Duan, Huixia Cui, Wenlu Zhang, Shan Wu

**Affiliations:** ^1^ College of Nursing, Jinzhou Medical University, Jinzhou, China; ^2^ Department of Radiation Oncology, The First Affiliated Hospital of Jinzhou Medical University, Jinzhou, China; ^3^ Department of Rheumatology, The First Affiliated Hospital of Jinzhou Medical University, Jinzhou, China

**Keywords:** chemotherapy, experiences, frailty, lung cancer, symptoms

## Abstract

**Objective:**

The aim of this study was to explore the symptoms and experiences of frailty in lung cancer patients treated with chemotherapy.

**Methods:**

Quantitative and qualitative research methods were implemented. A total of 302 patients aged > 18 years were recruited by convenience sampling method. Quantitative data were collected through the General Demographic Characteristics questionnaire, the Frailty Phenotype scale, the Cancer Fatigue Scale, the Hospital Anxiety and Depression Scale and the Pittsburgh Sleep Quality Index. Fourteen patients with a score of Frailty Phenotype scale ≥ 3 were drawn and their interviews were thematically analyzed.

**Results:**

The mean Frailty Phenotype score was (1.63±1.35), suggesting that most of the patients were in pre-frailty conditions. A total of 64 (21.2%) patients were non-frail, 168 (55.6%) patients were pre-frail, 70 (23.2%) patients were frail. The mean CFS, HADS scores, and PSQI scores were (26.86±8.93), (15.42±9.73), and (6.18±4.39), respectively. The Number of chemotherapy times was positively associated with frailty. Anxiety fatigue, depression and poor sleep quality positively correlated with frailty. The qualitative research showed four themes. Theme 1: the most reported symptoms of frailty were physical symptoms and psychological symptoms. Physical symptoms included fatigue, low physical activity, weight loss and poor sleep quality. Psychological symptoms included anxiety, depression and low social activities. Theme 2: frailty was mainly related to lung cancer and chemotherapeutic drugs, which can cause decreased appetite, constipation and altered taste. Theme 3: patients used bad coping strategies to manage the symptoms of frailty. Theme 4: the social support of patients was weak, especially regarding emotional support.

**Conclusion:**

The most frequent symptoms reported by lung cancer patients treated with chemotherapy were anxiety, fatigue, depression, low physical activity and poor sleep quality. Patients also complained of bad coping strategies and weak support. Medical staff should strengthen the management of frailty, aiming at improving the quality of life in lung cancer patients treated with chemotherapy.

## 1 Introduction

Frailty refers to a complex condition that is characterized by the decreased physiological function of multiple organs and systems, and this disease could increase the susceptibility of patients to stressors (1). Frailty categorizes individuals into three categories: non-frail individuals, pre-frail individuals and frail individuals (2). Frailty is a dynamic process and patients can shift between different frailty states (3, 4). The prevalence of frailty among community residents aged 65 and over is about 10% (5), however, a research revealed that the prevalence of frailty is between 9.1% and 59% in cancer patients (6). Frailty is associated with adverse outcomes, including falls, hospitalization and reduced life expectancy (7). Unlike many other cancers, lung cancer is associated with more severe symptoms, lower quality of life and poorer survival rate (8).

In 2020, approximately 2.21 million people were diagnosed with lung cancer (9). Almost half of lung cancer patients are 70-year-old or older (10). Lung cancer is the main cause of cancer death, and 1.80 million died from lung cancer (9). Depending on the type and stage, lung cancer is treated with surgery, chemotherapy, radiation therapy and/or a combination of these treatments. Around 73% of lung cancer patients need chemotherapy at least once in their lifetime (11). However, chemotherapy is frequently associated with myelosuppression, fatigue, loss of appetite, nausea, bowel issues, hair loss, mouth sores and/or psychological symptoms (12, 13). At the same time, chemotherapy produces a large amount of free radicals and induce oxidative stress, promoting premature aging and frailty (12, 14). Frailty has been identified as a predictor of postoperative complications, chemotherapy intolerance, disease progression and death (15). In a study by Wang et al (16), 1020 elderly lung cancer patients aged 60 years or older before receiving phase I chemotherapy were included to predict mortality and adverse effects of chemotherapy by constructing a frailty index based on routine laboratory data. The overall median survival rate was lower in frailty patients. Caring for frail cancer patients is a complex and stressful task, that may cause significant challenges to caregivers, including nurses.

Patients with lung cancer are frequently diagnosed at an advanced stage and when they are older (17, 18). Older people are more likely to be stimulated by tumors and treatments that can impair internal balance, triggering the onset of frailty, due to their reduced physiological reserves and increased susceptibility to disease (19). In addition, researchers discovered that frailty and tumorigenesis share inflammatory factors: IL-6 and TNF-α. Increased IL-6 levels are linked to frailty, and IL-6 increases the likelihood of lung cancer metastasis. TNF-α is an endogenous tumor promoter, and tumor TNF-α production is linked to a poor prognosis, hormonal response, and cachexia. TNF-α levels were found to be higher in frail patients by Joanna et al. (20–22). Furthermore, due to the negative effects of chemotherapy on patients, the incidence of frailty in lung cancer patients receiving chemotherapy may be higher. This study aimed at investigating physical and psychological symptoms in lung cancer patients treated with chemotherapy using a mixed-method approach, which can address the patients’ significant needs. This study would provide a reference for setting up new intervention strategies to alleviate frailty.

## 2 Methods

### 2.1 Study design and ethical considerations

**Figure 1:**
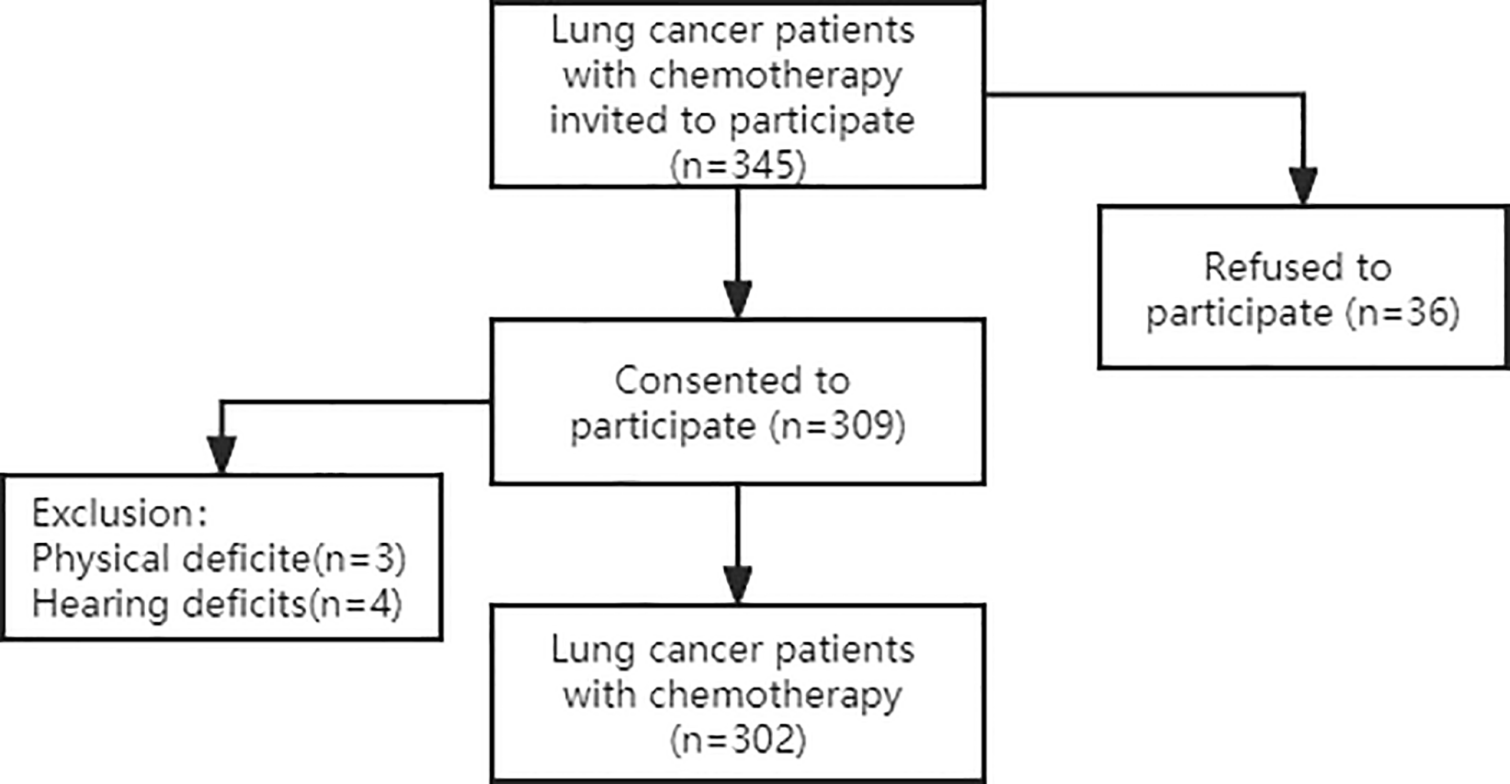
Flowchart of participants recruitment.

This was a convergent, parallel mixed-method study including quantitative and qualitative research methods. Participants were recruited from two hospitals, the First and Third Affiliated Hospitals of Jinzhou Medical University, between July 2021 and December 2021. 345 lung cancer chemotherapy patients were invited, 36 patients declined to participate, and 7 patients were excluded due to physical impairment to ensure the reliability of the reported data. Finally, a total of 302 lung cancer chemotherapy patients participated in a face-to-face structured questionnaire. Which are shown in **Figure 1**. The General Demographic Characteristics questionnaire, the Frailty Phenotype (FP) scale, the Cancer Fatigue Scale (CFS), the Hospital Anxiety and Depression Scale (HADS) and the Pittsburgh Sleep Quality Index (PSQI) were used.

### 2.2 Quantitative techniques

#### 2.2.1 Participants

A total of 302 lung cancer patients treated with chemotherapy were enrolled by convenience sampling between July 2021 and December 2021. Inclusion criteria were as follows: age ≥ 18 years old; diagnosis of lung cancer; treatment with chemotherapy; able to walk on their own or without the assistance of mobility devices and able to complete the walking test for this study. Exclusion criteria were as follows: concomitant diagnosis of mental disorders; inability to communicate clearly; involuntary participation in the study.

#### 2.2.2 The general demographic characteristics questionnaire

The study team designed the questionnaire after a thorough literature review. The questionnaire consists of several variables, including age, gender, residence, monthly income level, education level, familial history of cancer, grading and staging.

#### 2.2.3 The Frailty Phenotype scale

The FP scale is one of the most widely used tools to measure frailty (1). It consists of 5 items: exhaustion, weakness, slowness, low physical activity and unexplained weight loss, defined as a weight loss of more than 4.5 kg or 5% of body weight in one year. Exhaustion was captured using 2 items from the 20-item Centre for Epidemiological Studies Depression (CES-D) scale. Weakness was reflected by grip strength measured with a dynamometer on the dominant hand. Slowness was based on the time used to walk 15 seconds at the usual pace. Low physical activity consisted of weekly physical activity < 383 kcal for men and < 270 kcal for women. Patients were considered non-frail if no frailty components were present, pre-frail if one or two items were positive, frail if three or more items were positive.

#### 2.2.4 The cancer fatigue scale

The CFS was compiled by scholar Okuyama and reflected the nature of fatigue experienced by cancer patients (23). The CFS is a 15-item scale composed of 3 subscales (physical, affective and cognitive subscales). The scores range from 0 to 60 points. Items are rated on a four-point Likert scale (from 1 = never to 4 = most of the time).

#### 2.2.5 The hospital anxiety and depression scale

The HADS was compiled by Zigmond AS Which was used to detect states of depression and anxiety in the setting of an hospital medical outpatient clinic (24). It is a 14-question instrument, with each question being scored between 0 (no impairment) and 3 (severe impairment), with a maximum score of 21 for anxiety or depression.

#### 2.2.6 The Pittsburgh Sleep Quality Index

The PSQI was compiled by Buysee Which was a self-report questionnaire that assesses sleep quality over a 1-month time interval (25). The PSQI contains 19 self-rated questions grouped into seven component scores, including subjective sleep quality, sleep latency, sleep duration, habitual sleep efficiency, sleep disturbances, use of sleeping medications and daytime dysfunction. The total score ranges from 0 to 21.

#### 2.2.7 Data analysis

Continuous variables are expressed as mean ± standard deviation (SD) or median ± interquartile range (IQR, P75-P25), categorical variables are expressed as ratios and percentages (%). The one-way variance (F) was used to compare variables between groups. The Spearmon correlation analysis examined the relationship among frailty, fatigue, anxiety, depression and poor sleep quality. The data were analyzed using the SPSS 26.0 statistical software. A *p* value < 0.05 was considered statistically significant.

### 2.3 Qualitative techniques

#### 2.3.1 Participants

Participants were selected randomized drawn from quantitative study. The inclusion criteria were as follows: an FP score ≥ 3 points; willing to report the personal experience. Based on the principle of sample saturation, 14 lung cancer patients with chemotherapy were interviewed. Selecting the interview site on the health publicity classroom. Two patients were unable to walk, thus the interview was performed at bed side. The included patients were identified with a letter between A and N.

#### 2.3.2 Qualitative procedure and instruments

The objective of the qualitative research was to collect frailty symptoms and experiences. Fourteen patients were randomly selected from the quantitative study. The data were collected by face-to-face and semi-structured interviews. The interviews were recorded and focused on patients´ tone, patients’ appearance, facial expressions, and other non-verbal messages. The interview contained seven questions.

They were as follows: (1) Could you talk about any symptoms you have experienced after chemotherapy (such as physical, psychological or social symptoms)? (2) Did these symptoms impact your life? (3) What do you think about the causes of these symptoms? (4) Did these symptoms change over time? (5) How did you handle these symptoms? (6) Did you require special assistance for these symptoms? (7) Did you have other thoughts on frailty? The interview recordings were transcribed within 24 hours by the two interviewers.

## 3 Results

### 3.1 Characteristics of study participants

We enrolled 302 lung cancer patients treated with chemotherapy. A total of 183 participants (60.6%) were men, 119 (39.4%) were women. The mean age was (61.74±8.80) years. Around 48.0% of participants completed high schools. Approximately 48.3% of participants were covered by urban healthcare insurances. Number of chemotherapy times was 3(1,6.25), considering the TNM staging, the grade IV was predominant (61.3%).

### 3.2 The incidence of frail

**Figure 2:**
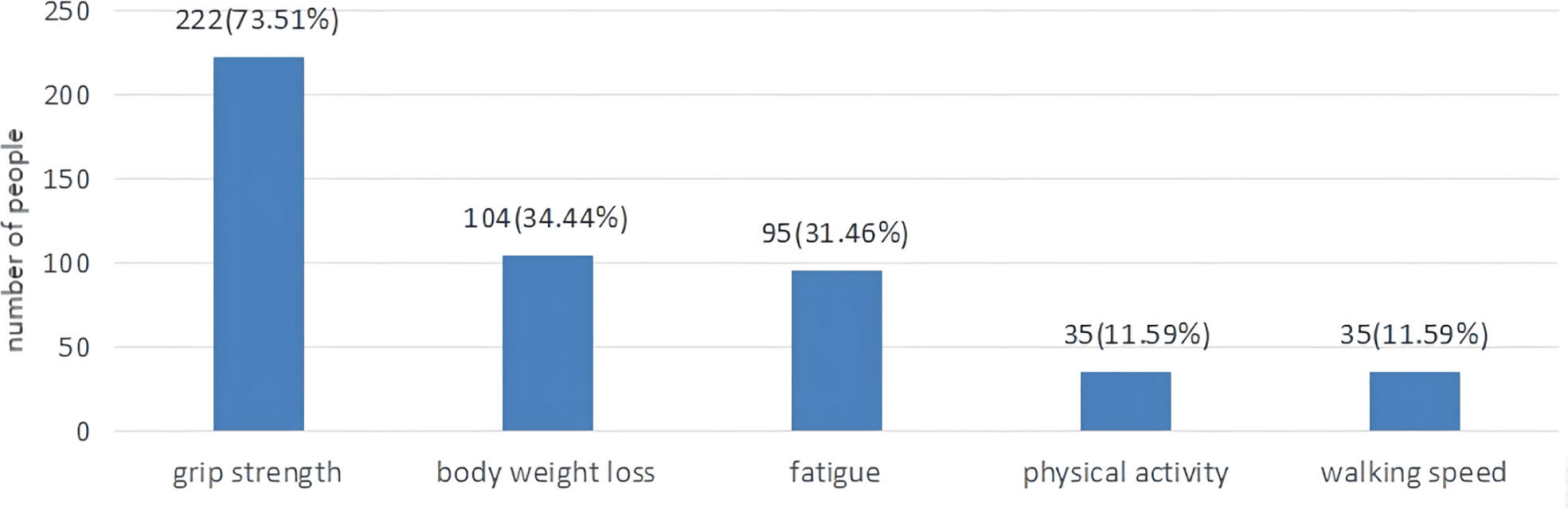
The proportion of lung cancer patients with chemotherapy according to five frailty phenotypes.

As displayed in **Table 1**, the mean FP score was (1.63±1.35). A total of 64 (21.2%) patients were non-frail, 168 (55.6%) patients were pre-frail, 70 (23.2%) patients were frail. As shown in **Figure 2**, 222 (73.5%) patients had a low grip strength, 104 (34.4%) patients reported an unexplained weight loss, 95 (31.5%) patients reported exhaustion, 35 (11.6%) patients reported low physical activity and 35 (11.6%) patients reported slowness.

**Table 1:** Frailty Phenotype score in lung cancer patients with chemotherapy (N=302).

Variables	Scores
FP total score	1.63 ± 1.35
Grip strength	0.74 ± 0.44
Body weight loss	0.34 ± 0.48
Fatigue	0.31 ± 0.47
Physical activity	0.12 ± 0.32
Walking speed	0.12 ± 0.32

### 3.3 The frailty status

When the frailty status was compared across age, gender, monthly income, TNM staging and the use of chemotherapeutics, there were significant differences (*p* < 0.05). The results are displayed in **Table 2**.

**Table 2:** Frailty status differences among demographic characteristics (N=302).

Variables		Mean ± SD or N%			F	P
		normal	prefrailty	frailty		
Age		58.11 ± 9.50	62.31 ± 8.47	63.68 ± 8.07	7.838	<0.001
Gender	Male	45	108	30	12.713	0.002
	Female	19	60	40		
Residence	City	37	107	51	3.436	0.179
	Urban	27	61	19		
Family history of cancer	Yes	12	26	12	0.382	0.826
	No	52	142	58		
Educational level	Primary or illiterate	11	35	19	6.706	0.349
	Junior high school	28	87	30		
	High school	16	35	13		
	University or above	9	11	8		
Medical insurance	No medical insurance	3	6	2	0.826	0.935
	Medical insurance for urban residents	28	83	34		
	Medical insurance for urban workers	33	79	34		
Monthly income,	<1000	2	8	3	13.073	0.042
	1000-3000	10	49	16		
	3000-5000	19	63	30		
	>5000	33	48	21		
Smoking history	Yes	37	96	30	4.541	0.103
	No	27	72	40		
TNM staging	I	9	10	3	25.434	<0.001
	II	7	7	4		
	III	17	56	7		
	IV	31	95	56		
Therapy method	Chemotherapy	36	76	24	10.823	0.212
	Chemotherapy + radiation therapy	4	24	13		
	Surgery + chemotherapy	15	39	20		
	Surgery + chemotherapy + radiation	5	11	3		
	Chemotherapy + others	4	18	10		
Transfer case	Yes	35	86	44	2.714	0.257
	No	29	82	26		
Number of chemotherapy times		3.79 ± 3.48	4.39 ± 4.33	7.47 ± 7.78	10.82	<0.001

### 3.4 Correlation analysis

The research shows that Number of chemotherapy times was positively associated with frailty (*r*=0.193, *P*<0.001). **Table 3** shows the mean CFS score (26.86±8.93). The highest scores related to physical fatigue and affective fatigue were (14.77±4.83) and (6.77±2.94), respectively. Fatigue was significantly and positively correlated with frailty (**Table 4**).

**Table 3:** Cancer Fatigue Scale scores in lung cancer patients with chemotherapy (N = 302).

Variables	Scores
CFS total score	26.86 ± 8.93
Physical fatigue	14.77 ± 4.83
Affective fatigue	6.77 ± 2.94
Cognitive fatigue	5.31 ± 2.35

**Table 4:** Correlation analysis among frailty and fatigue (N = 302).

Variables	Frailty	Grip strength	Body weight loss	Fatigue	Physical activity	Walking speed
CFS total score	0.585**	0.408**	0.342**	0.558**	0.398**	0.449**
Physical fatigue	0.543**	0.369**	0.339**	0.542**	0.343**	0.407**
Affective fatigue	0.565**	0.439**	0.318**	0.493**	0.370**	0.381**
Cognitive fatigue	0.399**	0.244**	0.206**	0.390**	0.344**	0.393**

** P<0.01.

**Table 5** illustrates the mean PSQI score (6.18±4.39). The highest scores related to sleep latency and daytime dysfunction were (1.71±1.15) and (1.45±1.08), respectively. The PSQI scores were significantly and positively correlated with frailty (**Table 6**).

**Table 5:** Pittsburgh Sleep Quality Index scores in lung cancer patients with chemotherapy (N = 302).

Variables	Scores
PSQI total score	6.18 ± 4.39
Subjective sleep quality	1.28 ± 0.87
Sleep latency	1.71 ± 1.15
Sleep duration	0.37 ± 0.84
Sleep efficiency	0.46 ± 0.88
Sleep disturbances	0.74 ± 0.61
Sleeping medication	0.17 ± 0.62
Daytime dysfunction	1.45 ± 1.08

**Table 6:** Correlation analysis among frailty and sleep quality (N = 302).

Variables	Frailty	Grip strength	Body weight loss	Fatigue	Physical activity	Walking speed
PSQI total score	0.405**	0.276**	0.327**	0.342**	0.271**	0.224**
Subjective sleep quality	0.336**	0.222**	0.280**	0.275**	0.217**	0.217**
Sleep latency	0.276**	0.201**	0.220**	0.296**	0.191**	0.173**
Sleep duration	0.231**	0.089	0.251**	0.132*	0.234**	0.11
Sleep efficiency	0.200**	0.084	0.240**	0.157**	0.176**	0.07
Sleep disturbances	0.387**	0.292**	0.212**	0.259**	0.242**	0.174**
Sleeping medication	0.08	0.039	0.099	0.095	0.02	0.037
Daytime dysfunction	0.477**	0.406**	0.303**	0.423**	0.251**	0.289**

** P<0.01.

The mean HADS score was (15.42±9.73), as shown in **Table 7**. The HADS scores were significantly and positively correlated with frailty (**Table 8**).

**Table 7:** Hospital Anxiety and Depression Scale in lung cancer patients with chemotherapy (N = 302).

Variables	Scores
HADS total score	15.42 ± 9.73
Anxiety	6.57 ± 4.56
Depression	8.85 ± 5.72

**Table 8:** Correlation analysis among frailty and anxiety and depression (N = 302).

Variables	Frailty	Grip strength	Body weight loss	Fatigue	Physical activity	Walking speed
HADS total score	0.491**	0.377**	0.306**	0.385**	0.361**	0.352**
Anxiety	0.410**	0.307**	0.251**	0.314**	0.337**	0.330**
Depression	0.508**	0.397**	0.320**	0.404**	0.346**	0.335**

** P<0.01

### 3.5 The qualitative research

Fourteen lung cancer patients using chemotherapy were interviewed. They were 6 men and 8 women, aged from 54 to 73 years old. The patients´ characteristics are shown in **Table 9**. We summarized four themes arising from the semi-structured interviews: symptoms of frailty, causes of frailty, coping strategies and medical support.

**Table 9:** Characteristics of participants (N = 14).

Number	Age	Gender	Histopathological type	TNM staging	Chemotherapy cycle	Course of the disease	The score of FP
A	65	Male	SCC	II	4	2	3
B	58	Female	Adenocarcinoma	IV	6	12	3
C	58	Female	Adenocarcinoma	IV	3	60	5
D	73	Male	SCC	IV	6	14	3
E	57	Male	Adenocarcinoma	IV	1	1	3
F	65	Female	SCC	IV	2	12	3
G	73	Female	Adenocarcinoma	IV	11	51	5
H	65	Female	Adenocarcinoma	IV	3	6	3
I	71	Female	SCLC	IV	9	30	5
G	60	Female	Adenocarcinoma	IV	6	9	3
K	59	Male	Adenocarcinoma	IV	9	12	3
L	63	Male	Adenocarcinoma	IV	8	12	4
M	54	Female	SCC	II	2	6	3
N	61	Male	Adenocarcinoma	IV	5	9	3

SCC, squamous cell carcinoma; SCLC, small cell lung cancer.

#### 3.5.1 Theme 1: Symptoms of frailty

##### 3.5.1.1 Sub-theme 1: Physiological symptoms

###### 3.5.1.1.1 Fatigue

All patients reported exhaustion, one of the main symptom of frailty. During chemotherapy, patients developed fatigue quickly and intensely. The interviewee A reported “I felt exhausted. I did not have strength in my arms and legs”. The interviewee B reported “I felt very tired and I could not do anything at home”. The interviewee H reported “I was fatigued at the beginning of chemotherapy”.

###### 3.5.1.1.2 Low physical activity

Considering physical activity, most activities were mild. interviewee D reported “At the beginning of chemotherapy, I did not want to take a walk because of my poor physical strength”. The interviewee I reported “I could do moderate or intense physical activity. From the fourth course of treatment, I felt pain in my legs which prevented me from doing any activities”.

###### 3.5.1.1.3 Body weight loss

Seven out of fourteen patients complained unexplained weight loss. The interviewee C reported “I lost ten pounds in the last two months, that is why I see the doctor”. The interviewee H reported “Since chemotherapy, I lost my appetite and 13 pounds”.

###### 3.5.1.1.4 Poor sleep quality

Seven out of fourteen patients complained poor sleep quality. The interviewee B reported “The quality of my sleep was poor, sometimes it took an hour or two to fall asleep”. The interviewee M reported “Since the illness, my sleep was not as good as before”.

##### 3.5.1.2 Sub-theme 2: Psychological symptoms

###### 3.5.1.2.1 Anxiety and depression

Four patients complained severe anxiety and depression. The interviewee F reported “I had surgery and I spent a lot of money, but I did not know whether the disease would be cured” The interviewee B reported “I have a terrible temper and my family hides illness, making me very worried”.

###### 3.5.1.2.2 Avoidant sociability

Twelve patients refused to see people other than family members in their daily lives and feared being labeled as cancer patients. The interviewee B reported “Please look at my hair, I do not like to meet other people”. The interviewee K reported “Ever since I got sick, I did not want to meet people and take walks”.

#### 3.5.2 Theme 2: Causes of frailty

Eight patients suggested that frailty might be related to the disease. The interviewee E reported “I think frailty is associated with lung cancer, because I have felt very tired and dull since they found something in my lung”. The interviewee K reported “There must be a relationship to the disease. This disorder makes me feel short of breath and tight in my chest”. Thirteen patients believed that frailty might be related to chemotherapeutic drugs. The Interviewee C reported “When I took targeted drugs, I did not feel any adverse reaction. When I started chemotherapy, I felt fatigued and I could not do anything”. Nine patients complained decreased appetite and constipation. The Interviewee K reported “I could not eat for a few days after chemotherapy”. Some patients developed symptoms of altered taste, pain and cardiac issues. The Interviewee C reported “I had a change of taste, sometimes there is a metallic taste drinking mineral water”. The Interviewee I reported “Since the chemotherapeutic drug was changed to paclitaxel, I feel pain all over my body and I cannot walk”.

#### 3.5.3 Theme 3: Coping strategies to reduce frailty

Eight patients reported bad coping strategies to reduce frailty. The Interviewee B reported “I did not know how to deal with frailty, I thought it as a regular part of chemotherapy”. Four patients complained passive responses to frailty. The interviewee C reported “I felt very fatigued when I returned home, I should be down and rest. Only a few days later, I felt a little better”. Only one Christian patient reported a positive response to frailty. The Interviewee G reported “When I was unfortunate, I found a quiet place to tell God. With that, my mood felt better”.

#### 3.5.4 Theme 4: Medical support

Patients reported a poor medical support, especially considering medical information and emotional support. The Interviewee I reported “Now, I hope doctors or nurses can tell me how to deal with the adverse reaction. When it happened, I was terrified”. Again, the Interviewee I reported “If the doctors or nurses could talk about my troubles, I would be pleased. But they are too busy and I feel embarrassed to bother them”.

## 4 Discussion

Frailty is a multidimensional concept used to predict outcomes in surgical and general medicine settings, but less information is available in cancer patients. This mixed-method study explored symptoms and experiences in lung cancer patients treated with chemotherapy. We showed that the mean FP score was (1.63±1.35), meaning that most of patients were pre-frail. The proportions of pre-frail and frail patients were 55.6% and 23.2%, respectively. Our rate was higher than a previous study, where the proportion of elderly cancer patients with frailty was 16.1% (26). This may due to the presence of comorbidities and higher age (median age, 61.74±8.80 years) in lung cancer patients (27). And some of the patients with lung cancer at diagnosis are cachectic with systemic inflammation and related weight loss, anemia, metabolic alterations that can determine a condition of frailty and systemic symptoms such as fatigue etc (28).

### 4.1 Demographic characteristics influencing frailty

The age of non-frail, pre-frail and frail patients was (58.11±9.50), (62.31±8.47) and (63.68±8.07) years, respectively. Similarly to a previous study (29), the age was positively associated with frailty. With increasing age, patients were more likely to have functional decline and increased susceptibility to frailty. The numbers of chemotherapeutic cycles in non-frail, pre-frail and frail patients were (3.79±3.48), (4.39±4.33) and (7.47±7.78), respectively. The number of chemotherapeutic cycles was positively associated with frailty. In accordance, the qualitative research found that the causes of frailty were mainly reported as related to the disease and chemotherapeutic drugs.

### 4.2 Frail symptoms in lung cancer patients treated with chemotherapy

The quantitative results showed that the mean CFS score was (26.86±8.93), while frailty was positively correlated with fatigue. The total scores of physical, emotional and cognitive fatigue were (14.77±4.83), (6.77±2.94) and (5.31±2.35), respectively. Similarly, to the qualitative research physical fatigue was one of the main symptoms of frailty reported by lung cancer patients treated with chemotherapy. During the interviews, patients complained fatigue after chemotherapy. Fatigue is often overlooked in lung cancer patients. Cancer-related fatigue was previously described as a distressing and persistent sense of physical, emotional and cognitive exhaustion unproportioned to recent physical activity (30). Nevertheless, frailty was described as a functional decline in physical strength and endurance (31). Frailty could contribute to fatigue, while fatigue might accelerate the occurrence and development of frailty. In accordance with our findings, previous surveys showed that fatigue and frailty were positively associated. Of interest, we showed that 222 (73.5%) patients reported a low grip strength and 104 (34.4%) patients reported an unexplained weight loss. A low grip strength is prevalent in lung cancer patients, but not easily detectable. Some patients only notice it when conducting moderate or intense physical activity. A total of 95 (31.5%) patients reported exhaustion. The CFS scores comprehensively assess subjective fatigue, while the FP scale evaluates the impact of fatigue on quality of life. Similarly, to the qualitative research, most lung cancer patients reported only mild physical activity. A previous study found that a lower physical activity increased the risk of frailty (32). Moreover, an intense physical activity reduced the FP scores and the Tilburg Frailty Indicator (TFI) scores (33). It would be preferable for lung cancer patients to increase their physical activity.

Regarding the PSQI scores, we showed that the mean value was (6.18±4.39). The sleep latency and daytime dysfunction scores were (1.71±1.15), and (1.45±1.08), respectively. The daytime dysfunction and grip strength scores were highly correlated in frail patients. The qualitative research revealed that patients usually have difficulty falling asleep and they wake up easily during the night. According to our findings, a poor sleep quality produced negative emotions such as anxiety and irritability. When poor sleep quality becomes frequent, it can cause diabetes mellitus, cardiovascular diseases, cancer and other disorders (34). Doctors and nursing staff should help and support patients to develop better sleep habits by doing more physical exercise and reducing lunch breaks. Considering the HADS scores, the mean value was (15.42±9.73). During the interviews, we found that some patients suffered from severe anxiety and depression. Some patients tried to interrupt the treatment and lost hope of living a healthy life. According to a previous study, patients treated with chemotherapy frequently reported symptoms such as anxiety, depression and inability to concentrate (35). The uncertainty about the future and the high economic burden might also increase the negative emotions, impacting on survival rates (36, 37). In lung cancer patients, psychological interventions relieved negative emotions, helping them to face the disease (38). Nurses and patients’ families should take a closer look to the psychological emotions of lung cancer patients.

The symptoms do not generally receive much attention from patients, resulting in a higher incidence of frailty. If symptoms like fatigue, weight loss, anxiety and/or depression are not detected early, they can predispose to frailty. A proper health education from healthcare professionals is advisable in lung cancer patients.

### 4.3 Analysis of the causes of frailty

According to qualitative and quantitative research. The causes of frailty in lung cancer chemotherapy patients were two main reasons: chemotherapy factors and non-chemotherapy factors. Although the exact causes of frailty are unclear, Previously, frailty was associated with age, malnutrition, sarcopenia, poor sleep quality, anxiety and depression (39–43). At the same time, factors associated with disease can contribute to frailty, such as inflammation or cachexia. We can refer to the above causes as non-chemotherapy factors. The use of chemotherapy medications, which shorten telomeres (12, 14) while increasing severe chemotherapy side effects like loss of appetite, discomfort, altered taste, and cardiotoxicity, is the most significant chemotherapeutic factor. Patients with lung cancer who are undergoing chemotherapy are at a significant risk of frailty due to illness and age, and chemotherapy increases this risk. Therefore, it is important to identify and intervene in risk factors for frailty before it occurs.

### 4.4 Coping strategies and medical support

The qualitative research found that lung cancer patients often manifested a negative approach to frailty, only a few of them used nutritional and physical exercise interventions. Our patients and their families believed that weakness, appetite loss, weight loss and fatigue were side effects associated with chemotherapy, without seeking any help from medical staff. Doctors and nurses should explore new interventions and innovative methods for health education, including education strategies against frail. The current intervention for frailty is multidisciplinary, including physical exercise and nutrition (44, 45). However, targeted interventions for lung cancer patients treated with chemotherapy have not yet been developed. Nurses may prepare informative material and/or music videos, or support the use of Internet in health education. Patients could learn that adverse manifestations associated with frailty can be prevented and relieved.

### 4.5 Study limitations

This study has also some limitations. Firstly, the lung cancer patients were enrolled only from two hospitals, from a single province. Secondly, the causality was difficult to establish. Therefore, further research is required to draw firmer conclusions. Finally, the inclusion criteria for this study did not specifically define the chemotherapy cycle, so if patients underwent chemotherapy, they were included in the study regardless of the cycle, which could have an effect on the findings. As a result, the following step will be to undertake more research to supplement the current study that will focus on how the chemotherapy cycle affects frailty.

## 5 Conclusions

Our study found that not only age and disease, but also chemotherapy was an important risk factor for frailty. Lung cancer patients treated with chemotherapy are highly frail. They often show exhaustion, low physical activity, poor sleep quality and other difficulties in daily living, including intolerance to chemotherapy and treatment discontinuation. Doctors and nurses should enhance the management of frailty symptoms and strengthen communication with patients through different interventions, including Internet, health knowledge lectures, telephone calls and continuing care. There is a possibility to explore novel frailty intervention methods by establishing a multidisciplinary team.

## Data availability statement

The raw data supporting the conclusions of this article will be made available by the authors, without undue reservation.

## Ethics statement

This study was approved by the ethics committee of Jinzhou Medical University. The participants provided their written informed consent to participate in this study. Written informed consent was obtained from the individual(s) for the publication of any potentially identifiable images or data included in this article.

## Author contributions

LD, WZ and HC contributed to conception and design of the study and made corrections to the first draft. LD and SW organized the database. LD wrote the first draft of the manuscript. WZ and HC Critical revisions to the paper for important intellectual content. All authors contributed to manuscript revision, read, and approved the submitted version.

## Acknowledgments

We acknowledge TopEdit (www.topeditsci.com) for the linguistic editing and proofreading during the preparation of this manuscript.

## Conflict of interest

The authors declare that the research was conducted in the absence of any commercial or financial relationships that could be construed as a potential conflict of interest.

## Publisher’s note

All claims expressed in this article are solely those of the authors and do not necessarily represent those of their affiliated organizations, or those of the publisher, the editors and the reviewers. Any product that may be evaluated in this article, or claim that may be made by its manufacturer, is not guaranteed or endorsed by the publisher.
